# Local Anesthesia at ST36 to Reveal Responding Brain Areas to *deqi*

**DOI:** 10.1155/2014/987365

**Published:** 2014-01-16

**Authors:** Ling-min Jin, Cai-juan Qin, Lei Lan, Jin-bo Sun, Fang Zeng, Yuan-qiang Zhu, Shu-guang Yu, Hai-yan Yin, Yong Tang

**Affiliations:** ^1^Life Sciences Research Center, School of Life Sciences and Technology, Xidian University, Xi'an, Shaanxi 710126, China; ^2^The 3rd Teaching Hospital, Chengdu University of Traditional Chinese Medicine, Chengdu, Sichuan 610075, China

## Abstract

*Background.* Development of non-*deqi* control is still a challenge. This study aims to set up a potential approach to non-*deqi* control by using lidocaine anesthesia at ST36. *Methods.* Forty healthy volunteers were recruited and they received two fMRI scans. One was accompanied with manual acupuncture at ST36 (DQ group), and another was associated with both local anesthesia and manual acupuncture at the same acupoint (LA group). *Results.* Comparing to DQ group, more than 90 percent *deqi* sensations were reduced by local anesthesia in LA group. The mainly activated regions in DQ group were bilateral IFG, S1, primary motor cortex, IPL, thalamus, insula, claustrum, cingulate gyrus, putamen, superior temporal gyrus, and cerebellum. Surprisingly only cerebellum showed significant activation in LA group. Compared to the two groups, bilateral S1, insula, ipsilateral IFG, IPL, claustrum, and contralateral ACC were remarkably activated. *Conclusions.* Local anesthesia at ST36 is able to block most of the * deqi* feelings and inhibit brain responses to *deqi*, which would be developed into a potential approach for non-*deqi* control. Bilateral S1, insula, ipsilateral IFG, IPL, claustrum, and contralateral ACC might be the key brain regions responding to *deqi*.

## 1. Introduction

*deqi*, also called needle sensation, refers to the sensations of soreness, numbness, fullness, heaviness, and so forth around the acupoints of patients when the needle is inserted to a certain depth. At the same time, the operator may feel heaviness or tension around the needle. As one of the most classic and important concepts originated from Neijing (The Yellow Emperor's Classic of Internal Medicine), *deqi* has drawn increasing attention of researchers recently [1–5]. The studies on *deqi* mainly focused on four directions: (1) identifying the relationship between *deqi* and acupuncture efficacy [6–10]; (2) quantifying the *deqi* sensations and making *deqi *visualization and objectification [11–13]; (3) investigating the influence factors on *deqi* such as insertion site, insertion depth, puncture manipulation and needle retaining time, and body position [14–19]; (4) exploring the mechanisms of *deqi* [20–31].

Whatever direction of investigations on *deqi*, it is essential to establish an appropriate non-*deqi* control. To date, several kinds of sham acupuncture strategies have been employed as non-*deqi* control, which include on-invasive placebo stimulation (Von Frey, Streitberger Needle, etc.) at the same acupuncture point or nonacupuncture point [7, 29, 31], superficial needling at the same acupuncture point or nonacupuncture point [9, 32], or needling at the nonacupuncture point or acupoint unrelated to the research purpose [20, 22, 23, 33].

However, clinical and neuroimaging studies demonstrated that acupuncture feelings were unable to be comprehensively inhibited by sham acupuncture [33, 34]. Therefore, development of non-*deqi* control is still a challenge. Giving the fact that both local anesthetic at the acupoint and general anesthesia had been applied to explore the mechanism of acupuncture in previous studies [35–38], we proposed that local anesthesia would be a potential strategy to be used as an approach of non-*deqi* control. Based on this idea, we set up a non-*deqi* control of acupuncture using local lidocaine anesthesia at acupoint and evaluating with the scoring of subjects' feelings and performed fMRI scanning to obtain the changed brain areas.

## 2. Methods

### 2.1. Subjects

Forty healthy, right-handed, adult volunteers (20 females and 20 males, ages ranged from 22 to 25 years) were recruited in this study. Prior to participation, all subjects provided written informed consent. They were also screened to assure their safety and compatibility with MRI recording and eliminate those with history of head trauma, chronic pain, psychiatric and neurological disorders, or other serious illness within 1 month.

### 2.2. Experimental Design

Each subject was trained to express his feelings about *deqi* correctly and clearly through acupuncturing at right Zusanli (ST36). If the subject rated at least one of single sensation (except sharp pain) at 4 (moderate intensity) or greater using a 10-points visual analogue scale (VAS), he would be included. Then at least 24 hours later, every included participant received two separate fMRI scans which were required in DQ or LA group, respectively. In DQ group, the subjects received fMRI scan and acupuncture stimulation simultaneously. In LA group, they underwent local anesthesia at ST36 firstly, then received scan and acupuncture intervention 5 min later. The two scans were randomly given at least 6 hours intervals. During the scanning, all subjects were instructed to keep supine position, head motionless, and eyes closed; soundproof earplugs were used to block noise. After each scan run, they were asked to finish a sensory questionnaire regarding the type and intensity of feelings they experienced during the scan. All acupuncture manipulations were performed by the same licensed acupuncturist.

#### 2.2.1. Acupuncture Procedures

**Figure 1:**
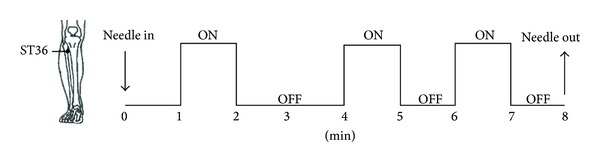
Experimental paradigm of acupuncture run.

In both DQ and LA group, the acupuncture intervention was employed at right Zusanli (ST36) by perpendicularly inserting 20 mm deep with sterilized disposable stainless steel acupuncture needles (0.25 × 40 mm, Suzhou Medical Supplies Factory Co. Ltd. China). The entire stimulating process lasted for 8 minutes. During 8 minutes, ON and OFF two states were designed and each total duration was 3 and 5 minutes, respectively (Figure 1). In the ON state, the inserted needle was rotated with moderate reinforcing and reducing method (twisting 60 times/min) for 1 min to generate *deqi*, while in the OFF state, the inserted needle was retained into the ST36 without rotation.

#### 2.2.2. Local Anesthesia Intervention

The local anesthesia at ST 36 was carried out to the subcutaneous depth of 20 mm by infiltrating with 2 mL (5 mL :  0.1 g) lidocaine.

#### 2.2.3. *deqi *Measurement

After removing the needle, subjects were asked to quantify their stimulating sensations including soreness, numbness, heaviness, fullness, dull or sharp pain, warmth, and coolness by VAS. The VAS was scaled as follows: 0: no sensation; 1–3: mild; 4–6: moderate; 7-8: strong; 9: severe; 10: unbearable. In DQ group, only subjects that rated at least one of the single sensations (except sharp pain) at 4 or greater were enrolled. Only subjects that rated each single sensation less than 1 were included in LA group.

#### 2.2.4. fMRI Scanning and Analysis

Imaging data were collected from a 3T Siemens scanner (Allegra, Siemens Medical System) at the Huaxi MR Research Center, West China Hospital of Sichuan University, Chengdu, China. A standard birdcage head coil was used, along with restraining foam pads to minimize head motion and diminish scanner noise. Thirty axial slices (FOV = 240 mm × 240 mm, matrix = 64 × 64, thickness = 5 mm) parallel to the AC-PC plane covering the whole brain were obtained using a T2*∗*-weighted single-shot, gradient-recalled echo planar imaging (EPI) sequence (TR = 2,000 ms, TE = 30 ms, flip angle = 90°). The scan covered the entire brain including the cerebellum and brainstem. After the functional run, high-resolution structural information on each subject was acquired using 3D MRI sequences with a voxel size of 1 mm^3^ for anatomical localization (TR = 2.7 s, TE = 3.39 ms, matrix = 256 × 256, FOV = 256 mm × 256 mm, flip angle = 7°, in-plane resolution = 1 mm × 1 mm, slice thickness = 1 mm).

Preprocessing and statistical analysis were performed using the Statistical Parametric Mapping software (SPM5, http://www.fil.ion.ucl.ac.uk/spm). Preprocessing of the functional images was composed of the following steps: dropping the first 5 time points; slice time correction; and three-dimensional motion correction. Then spatially smoothed was performed using a 6 mm full-width-at-half maximum (FWHM). After that, the time-series from each voxel was high-pass filtered (1/235-Hz cutoff). Subsequently, the preprocessed fMRI data for each subject was submitted for fixed-effects model analyses using the general linear model (GLM) performed at each voxel across the whole brain. After acquiring the contrast images, individual level analyses were accomplished and statistical parametric maps for the *t* statistics (spmT) were generated for each contrast image. At the group level, the random-effects model analysis was performed based on inference images (i.e., *t*-test for contrast images) from the individual level analysis. The group results of one sample *t*-test for each group were listed at *P* < 0.05, FWE (the DQ group |*t* | >6.14, the LA group |*t* | >5.86), and a minimum cluster size of 5 voxels. The different BOLD responses between the DQ group and the LA group were explored in 20 subjects (self-control) based on a paired *t*-test at *P* < 0.0001, uncorrected (|*t*| > 4.16), and a minimum cluster size of 5 voxels. Then, the significant regions of the paired *t*-test were defined as the regions of interests (ROIs). In each ROI, the BOLD response changes were extracted and correlated with the *deqi* score changes of the subjects who were included in paired *t*-test.

## 3. Results

Of 40 recruited volunteers, 5 subjects were eliminated because of slight *deqi* sensations (all the *deqi* scores less than 4) in training experiment. Consequently, 35 volunteers participated in the following scans. During scanning process, 4 subjects were also eliminated because of slight needling sensations in DQ group and 5 subjects were eliminated due to some degree of sensation retaining (one of the single *deqi* score more than 1) in LA group. When performing the data analysis, 4 subjects in DQ group and 2 subjects in LA group were excluded because larger amount of head motion had happened during scanning. Finally, 27 subjects and 28 subjects were, respectively, included in DQ group and LA group; 20 subjects in both DQ group and LA group were involved as self-control.

### 3.1. The *deqi *Scores Were Reduced by Local Anesthesia at ST36

The average scores of *deqi* sensations in each group are as follows (Table 1). The score of coolness in DQ group and warmth in LA group did not conform to normal distribution. Except coolness and warmth, other sensations were significantly reduced more than 90 percent by local anesthesia.

### 3.2. The Brain Responses Were Deleted by Local Anesthesia at ST36

**Figure 2:**
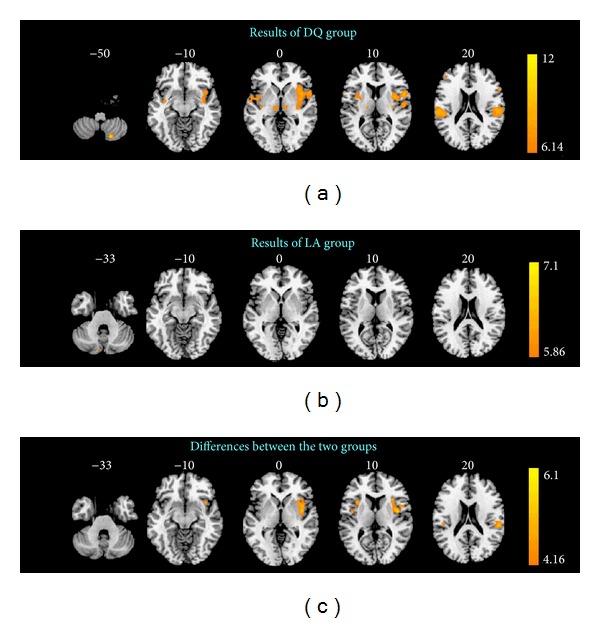
Group-level BOLD responses for both (a) DQ and (b) LA groups (*P* < 0.05, FWE) and (c) the differences between them (*P* < 0.0001, uncorrected).

Figure 2(a) showed group activations and deactivations of the DQ group evoked by acupuncture stimulation at ST36. The remarkable activated areas included bilateral inferior frontal gyrus (IFG), precentral gyrus (primary motor area, M1, supplementary motor area, SMA), postcentral gyrus (S1), inferior parietal lobule (IPL), thalamus, insula, anterior cingulate gyrus (ACC), claustrum, putamen, superior temporal gyrus (STG), midbrain and cerebellum, ipsilateral (right) transverse temporal gyrus (TTG), and contralateral (left) middle frontal gyrus (MFG) (FWE, *P* < 0.05) (Table 2). Based on this threshold, no deactivated areas were found.

Figure 2(b) showed the brain responses which were deleted by local anesthesia. Under this condition, only contralateral cerebellum was activated (FWE, *P* < 0.05). It was interesting to note that at group level few regions responding to acupuncture stimulation were found, but at individual level lots of BOLD signal intensity changes displayed. Considering the possibility that different *deqi* intensity elicited different cortical activation, we divided the LA group into three subgroups based on the sum score of *deqi* (non-*deqi* group: 10 subjects, averaged sum score of *deqi* was 0; *deqi* group A: 9 subjects, averaged sum score of *deqi* was 0.81 ± 0.29;* deqi* group B: 9 subjects, averaged sum score of *deqi* was 2.17 ± 0.47). We found no different activation between the intersubgroup results using *t*-test (*P* > 0.01, uncorrected for all voxels, figure not shown).

Figure 2(c) presented results between these two groups. Controlling for BOLD response to LA group, we found that increased *deqi* sensations were associated with activation in bilateral S1, insula, ipsilateral IFG, IPL, claustrum, and contralateral ACC (*P* < 0.0001, uncorrected) (Table 2). No obvious difference was demonstrated between the deactivated regions of the two groups. All of these activated areas were defined as ROIs, and each ROI BOLD response changes had no significant correlations with each single sensation score changes or sum score changes of *deqi* between the two groups (*P* > 0.01, figure not shown).

## 4. Discussion

To our knowledge, this study was the first to establish the non-*deqi* control using lidocaine anesthesia at the acupuncture point ST36. Although the data (Table 1) showed us more than 90 percent other than 100% of *deqi* sensations were inhibited, it still should be able to provide a strong evidence for supporting that lidocaine anesthesia at the acupuncture point would be a promising approach for non-*deqi* control because all responding brain regions seen in DQ group (Figure 2(a)) disappeared in LA group (Figure 2(b)). In other words, retaining intensity of *deqi *is not efficient to give rise to brain response. In view of brain response to *deqi*, lidocaine anesthesia at acupoint would be an alternative to be used as a new non-*deqi* control.

By means of this control, we found that the activated brain regions were bilateral S1, insula, ipsilateral IFG, IPL, claustrum, and contralateral ACC (Figure 2(c)). These results were quite different from those in previous studies. In this study, the number of activated brain regions was remarkably reduced. However, more activated brain areas including the secondary somatosensory cortex (S2), the cerebellum, the thalamus, the primary motor cortex (M1), the superior temporal gyrus (STG), the visual cortices, the premotor and supplementary motor cortex ((pre)SMA), the basal ganglia, and the medial temporal gyrus (MTG) could be found to be activated in previous studies [29, 30, 39, 40] apart from those activated brain areas in our study. To some degree, current activated brain areas (bilateral S1, insula, ipsilateral IFG, IPL, claustrum and contralateral ACC) might be regarded as the net activated brain regions response to *deqi, *which dominantly associated with sensory and emotion. It also implied that the components of *deqi* mainly include perception and emotion.

However, it needs to perform more to confirm the results with more considerations. Firstly, except applying VAS as a tool to evaluate *deqi*, we should choose more different scales to evaluate *deqi*. Additionally, the sensations of *deqi* would be felt by operator and subjects at the same time based on traditional concepts of acupuncture. So,what would happen at operator's side after local anesthesia administering at acupoint is also waiting for more researches to be carried out.

## 5. Conclusions

The application of local anesthesia at ST36 is able to block most of the *deqi* feelings and inhibit brain responses to *deqi*, which would be a potential and promising approach for non-*deqi* control. Bilateral S1, insula, ipsilateral IFG, IPL, claustrum, and contralateral ACC might be the key brain regions responding to *deqi*.

## Figures and Tables

**Table 1:** Differences of *deqi* sensation VAS scores between DQ group and LA group.

*deqi* sensation	DQ group		LA group		Decreased ratio (%)
	Mean ± SD	95% CI	Mean ± SD	95% CI	
Sourness	2.96 ± 2.64	1.92–4.01	0.17 ± 0.43	0.01–0.34	94
Numbness	2.29 ± 2.55	1.28–3.30	0.08 ± 0.30	−0.03–0.20	96
Fullness	6.23 ± 1.89	5.48–6.98	0.46 ± 0.57	0.24–0.68	92
Spread	3.26 ± 2.78	2.16–4.36	0.12 ± 0.34	−0.01–0.25	96
Dull pain	1.46 ± 2.55	0.45–2.47	0.02 ± 0.08	−0.01–0.05	98
Sharp pain	1.16 ± 1.37	0.62–1.70	0.03 ± 0.12	−0.01–0.08	97
Coolness	—	—	0.07 ± 0.26	−0.03–0.17	—
Warmth	0.096 ± 0.50	−0.10–0.29	—	—	—

VAS: visual analogue scale; SD: standard deviation; CI: confidence interval.

**Table 2:** Significant BOLD response changes of DQ group and intergroup.

Regions	BA		DQ group (*P* < 0.05, FWE)					DQ versus LA (*P* < 0.0001, uncorr.)				
			Talairach			*t *	Voxels	Talairach			*t *	Voxels
			*x *	*y *	*z *			*x *	*y *	*z *		
Inferior frontal gyrus	9/47	L	−56	13	30	7.89	7					
		R	59	15	16	7.77	30	45	14	−3	5.08	13
Middle frontal gyrus	46	L	−42	45	23	7.92	16					
Precentral gyrus	4/6	L	−56	6	11	6.56	6					
		R	53	12	8	8.19	21					
Postcentral gyrus	2/40	L	−59	−25	18	9.47	50	−56	−21	43	4.68	5
		R	53	−25	21	8.28	48	56	−28	21	5.38	21
Inferior parietal lobule	40	L	−65	−31	29	10.05	56					
		R	62	−28	29	9.86	49	53	−28	24	4.88	7
Thalamus		L	−6	−17	4	7.80	47					
		R	6	−17	6	7.34	37					
Insula	13/40	L	−48	−28	18	8.94	43	−33	15	10	4.75	10
		R	42	8	−5	8.60	79	42	−2	8	5.77	53
Claustrum		L	−33	6	5	7.05	9					
		R	36	0	3	8.74	26	33	12	2	5.16	17
Anterior cingulate gyrus	24/32	L	−3	2	41	6.45	5	−6	2	39	4.65	6
		R	3	2	44	6.82	7					
Putamen		L	−27	0	8	6.52	7					
		R	30	−17	6	7.71	19					
Superior temporal gyrus	22/38	L	−59	−28	15	7.92	35					
		R	53	−11	9	8.20	34					
Transverse temporal gyrus	41/42	R	53	−17	12	6.93	5					
Midbrain		L	−9	−18	−2	6.77	10					
Cerebellum	Posterior	L	−18	−66	−40	6.50	6					
		R	18	−75	−39	11.50	40					

Note: the coordination of voxel with the maximal *t* within each region is listed.

## References

[1] Kong J, Gollub R, Huang T (2007). Acupuncture *deqi*, from qualitative history to quantitative measurement. *Journal of Alternative and Complementary Medicine*.

[2] Hui KK, Nixon EE, Vangel MG (2007). Characterization of the “*deqi*” response in acupuncture. *BMC Complementary and Alternative Medicine*.

[3] Chen S, Guo S, Marmori F (2013). Appraisal of the *deqi* concept among contemporary Chinese acupuncturists. *Evidence-Based Complementary and Alternative Medicine*.

[4] Yang XY, Shi GX, Li QQ, Zhang ZH, Xu Q, Liu CZ (2013). Characterization of *deqi* sensation and acupuncture effect. *Evidence-Based Complementary and Alternative Medicine*.

[5] Yuan HW, Ma LX, Qi DD, Zhang P, Li CH, Zhu J (2013). The historical development of concept from classics of traditional Chinese medicine to modern research: exploitation of the connotation of in Chinese medicine. *Evidence-Based Complementary and Alternative Medicine*.

[6] Berman BM, Lao L, Langenberg P, Lee WL, Gilpin AMK, Hochberg MC (2004). Effectiveness of acupuncture as adjunctive therapy in osteoarthritis of the knee. A randomized, controlled trial. *Annals of Internal Medicine*.

[7] Xiong J, Liu F, Zhang M, Wang W, Huang G (2012). *De-qi*, not psychological factors, determines the therapeutic efficacy of acupuncture treatment for primary dysmenorrhea. *Chinese Journal of Integrative Medicine*.

[8] Xu SB, Huang B, Zhang CY (2013). Effectiveness of strengthened stimulation during acupuncture for the treatment of Bell palsy: a randomized controlled trial. *Canadian Medical Association Journal*.

[9] Witt C, Brinkhaus B, Jena S (2005). Acupuncture in patients with osteoarthritis of the knee: a randomised trial. *The Lancet*.

[10] Spaeth RB, Camhi S, Hashmi JA (2013). A longitudinal study of the reliability of acupuncture *deqi* sensations in knee osteoarthritis. *Evidence-Based Complementary and Alternative Medicine*.

[11] White P, Bishop F, Hardy H (2008). Southampton needle sensation questionnaire: development and validation of a measure to gauge acupuncture needle sensation. *Journal of Alternative and Complementary Medicine*.

[12] Pach D, Hohmann C, Lüdtke R, Zimmermann-Viehoff F, Witt CM, Thiele C (2011). German translation of the southampton needle sensation questionnaire: use in an experimental acupuncture study. *Forschende Komplementarmedizin*.

[13] Kim Y, Park J, Lee H, Bang H, Park H (2008). Content validity of an acupuncture sensation questionnaire. *Journal of Alternative and Complementary Medicine*.

[14] Zhou K, Fang J, Wang X (2011). Characterization of *de qi* with electroacupuncture at acupoints with different properties. *Journal of Alternative and Complementary Medicine*.

[15] Benham A, Phillips G, Johnson MI (2010). An experimental study on the self-report of acupuncture needle sensation during deep needling with bi-directional rotation. *Acupuncture in Medicine*.

[16] Itoh K, Minakawa Y, Kitakoji H (2011). Effect of acupuncture depth on muscle pain. *Chinese Medicine*.

[17] Loyeung BY, Cobbin DM (2013). Investigating the effects of three needling parameters (manipulation, retention time, and insertion site) on needling sensation and pain profiles: a study of eight deep needling interventions. *Evidence-Based Complementary and Alternative Medicine*.

[18] Park JJ, Akazawa M, Ahn J (2011). Acupuncture sensation during ultrasound guided acupuncture needling. *Acupuncture in Medicine*.

[19] Chen XZ, Yang YK, Yang J (2013). Acupuncture *deqi* intensity and propagated sensation along channels may, respectively, differ due to different body positions of subjects. *Evidence-Based Complementary and Alternative Medicine*.

[20] Kuo T, Lin C, Ho F (2004). The soreness and numbness effect of acupuncture on skin blood flow. *American Journal of Chinese Medicine*.

[21] Takamoto K, Hori E, Urakawa S (2010). Cerebral hemodynamic responses induced by specific acupuncture sensations during needling at trigger points: a near-infrared spectroscopic study. *Brain Topography*.

[22] Yin CS, Park H, Kim S (2010). Electroencephalogram changes according to the subjective acupuncture sensation. *Neurological Research*.

[23] Huang S, Chen G, Lo H, Lin J, Lee Y, Kuo C (2005). Increase in the vagal modulation by acupuncture at Neiguan point in the healthy subjects. *American Journal of Chinese Medicine*.

[24] Fang J, Wang X, Liu H (2012). The limbic-prefrontal network modulated by electroacupuncture at CV4 and CV12. *Evidence-based Complementary and Alternative Medicine*.

[25] Fang J, Jin Z, Wang Y (2009). The salient characteristics of the central effects of acupuncture needling: limbic-paralimbic-neocortical network modulation. *Human Brain Mapping*.

[26] Hui KK, Liu J, Makris N (2000). Acupuncture modulates the limbic system and subcortical gray structures of the human brain: evidence from fMRI studies in normal subjects. *Human Brain Mapping*.

[27] Hui KK, Liu J, Marina O (2005). The integrated response of the human cerebro-cerebellar and limbic systems to acupuncture stimulation at ST 36 as evidenced by fMRI. *NeuroImage*.

[28] Wang X, Chan ST, Fang J (2013). Neural encoding of acupuncture needling sensations: evidence from a FMRI study. *Evidence-Based Complementary and Alternative Medicine*.

[29] Napadow V, Dhond RP, Kim J (2009). Brain encoding of acupuncture sensation—coupling on-line rating with fMRI. *NeuroImage*.

[30] Sun J, Zhu Y, Jin L (2012). Partly separated activations in the spatial distribution between *de-qi* and sharp pain during acupuncture stimulation: an fMRI-based study. *Evidence-Based Complementary and Alternative Medicine*.

[31] Chen JR, Li GL, Zhang GF, Huang Y, Wang SX, Lu N (2012). Brain areas involved in acupuncture needling sensation of *deqi*: a single-photon emission computed tomography (SPECT) study. *Acupuncture in Medicine*.

[32] Brinkhaus B, Ortiz M, Witt CM (2013). Acupuncture in patients with seasonal allergic rhinitis: a randomized trial. *Annals of Internal Medicine*.

[33] Huang W, Pach D, Napadow V (2012). Characterizing acupuncture stimuli using brain imaging with fMRI—a systematic review and meta-analysis of the literature. *PLoS ONE*.

[34] Vickers AJ, Cronin AM, Maschino AC (2012). Acupuncture for chronic pain: individual patient data meta-analysis. *Archives of Internal Medicine*.

[35] Chu H, Chang C, Khu XL, Yang LF (1973). Peripheral afferent pathway for acupuncture analgesia. *Science in China Series A-Mathematics*.

[36] Dundee JW, Ghaly G (1991). Local anesthesia blocks the antiemetic action of P6 acupuncture. *Clinical Pharmacology and Therapeutics*.

[37] Wang S, Constable RT, Tokoglu FS, Weiss DA, Freyle D, Kain ZN (2007). Acupuncture-induced blood oxygenation level-dependent signals in awake and anesthetized volunteers: a pilot study. *Anesthesia and Analgesia*.

[38] Schlünzen L, Vafaee MS, Cold GE (2007). Acupuncture of LI-4 in anesthetized healthy humans decreases cerebral blood flow in the putamen measured with positron emission tomography. *Anesthesia and Analgesia*.

[39] Beissner F (2011). Functional magnetic resonance imaging studies of acupuncture mechanisms: a critique. *Focus on Alternative and Complementary Therapies*.

[40] Chae Y, Chang DS, Lee SH (2013). Inserting needles into the body: a meta-analysis of brain activity associated with acupuncture needle stimulation. *Journal of Pain*.

